# Pathogenesis of cerebral amyloid angiopathy caused by chaotic glymphatics—Mini-review

**DOI:** 10.3389/fnins.2023.1180237

**Published:** 2023-04-11

**Authors:** Forshing Lui, Jessa Alcaide, Stella Knowlton, Michael Ysit, Ning Zhong

**Affiliations:** ^1^Department of Clinical Sciences, California Northstate University College of Medicine, Elk Grove, CA, United States; ^2^Department of Neurology, Kaiser Permanente Sacramento Medical Center, Sacramento, CA, United States

**Keywords:** cerebral amyloid angiopathy, glymphatics, aquaporin-4, Aβ clearance, astrocytes, parenchymal border macrophages

## Abstract

Cerebral amyloid angiopathy (CAA) is a common cause of lobar intracerebral hemorrhage in the elderly. It is also associated pathologically with Alzheimer’s disease (AD). Both CAA and AD share similar pathology of deposition amyloid beta fibrils (Aβ). Aβ is deposited mainly in the neurites in AD and vascular walls in CAA. Aβ is formed inside the brain parenchyma from the amyloid precursor protein. It is easier to understand how Aβ is deposited in the cerebral neurites in AD. However, the pathogenesis of CAA is still largely unknown. It is difficult to understand or visualize how Aβ fibrils formed inside the brain can be deposited against the cerebral perfusion pressure to be deposited in the cerebral and meningeal arterial walls. We encountered an unusual clinical case of acute aneurysmal subarachnoid hemorrhage which was followed after a few years with localized CAA involving mainly the sites of the subarachnoid hemorrhage. We reviewed the formation of Aβ and postulated how the Aβ fibrils are transported retrogradely toward the cerebral arteries and deposited in the arterial walls resulting in the final pathology of CAA. There is a clear disturbance of the glymphatic system, the aquaporin-4 channel, and the parenchymal border macrophages.

## Introduction

Cerebral amyloid angiopathy (CAA) is a common cause of lobar intracerebral hemorrhage in the elderly. It is also strongly associated with cognitive dysfunction and Alzheimer’s disease (AD). Pathologically, amyloid beta fibrils (Aβ) generated within the brain parenchyma deposit and accumulate within the walls of small to medium-sized blood vessels and capillaries in the brain parenchyma and leptomeninges. The diagnosis can be made via pathology or by the presence of strict criteria (Boston Criteria) (Charidimou et al., 2022) for lobar cerebral microbleeds. Interestingly, the prevalence of CAA confirmed by pathological diagnosis is threefold higher that by the Boston Criteria (23 vs. 7%) (Jäkel et al., 2022). The prevalence is 5–7% in cognitively normal elderly, 19–24% in patients with intracerebral hemorrhage, and 50–57% in patients with lobar intracerebral hemorrhage (Jäkel et al., 2022). CAA and AD share the common pathology of Aβ deposition in the central nervous system (CNS), and both conditions appear to result from impaired Aβ clearance from the brain (Greenberg et al., 2020). Impaired clearance of Aβ from the brain is easily understood, however, the deposition of Aβ in the arterial walls of the brain would require an additional step of movement. The Aβ fibrils from the brain must move toward the parenchymal arteries, opposite of the pressure and flow gradient out of the brain. This is the main reason why the pathogenesis of CAA is still largely unknown. With this review, we will try to analyze how the Aβ is formed, how it is normally cleared, and finally how it can be deposited in the arterial walls by using the available pieces of evidence.

## Formation of Aβ

Aβ deposition in the brain parenchyma is critical in the initiation and progression of AD. Its deposition in the brain vascular walls is the cause and hallmark of CAA. Aβ is generated by sequential cleavage of the amyloid precursor protein by the β- and γ- secretases. There are two main forms of Aβ, Aβ_40_, and Aβ_42_. Aβ_40_ is the more soluble form whereas Aβ_42_ is the insoluble form. Therefore it is easily understandable that parenchymal deposits in AD consist mainly of the insoluble Aβ_42_ whereas the predominant vascular wall deposit in CAA is the soluble Aβ_40_ (Ghiso et al., 2010). The excessive accumulation of Aβ in AD and CAA is caused either by excessive production, such as Down’s syndrome, or reduced clearance, such as in most cases of sporadic AD or CAA. The formation of Aβ, its toxicity, and pathogenesis in AD are presented clearly in a review by Sun et al. (2015).

## Pathways for clearance of Aβ

Protein solutes and metabolic wastes are cleared by the lymphatics in the peripheral tissues of our body, except for the CNS where there are no lymphatics in the usual understanding of anatomy. In order for solutes and metabolites to be cleared from the brain, they have to enter the brain’s interstitial fluid (ISF) space. From there, they would be moved to the cerebrospinal fluid (CSF) space and then cleared to the systemic lymphatic or vascular system. There are two functionally discrete spaces surrounding the blood vessels of the brain. These are the perivascular and paravascular spaces that move the solutes in opposite directions (Bacyinski et al., 2017).

The perivascular space lies within the tunica media of the penetrating cerebral arteries, between the middle layer of the basement membrane and the vascular smooth muscle cells. The solutes enter this space and are cleared from the brain by diffusion in the CSF or draining directly into cervical lymphatics (Morris et al., 2016; Ueno et al., 2016). This pathway is also called intramural arterial drainage.

The paravascular space (Virchow-Robin space, VR space) of the penetrating cerebral arteries contains CSF. The CSF then enters the brain parenchyma and mixes with ISF. The CSF, along with ISF moves toward the venous paravascular space where the solutes are removed from the brain through a convective flow process. This flow dynamics process was revolutionized by the work done by Iliff et al. (2012) and Iliff and Nedergaard (2013) on rodents. They created the term “glymphatics” to describe the flow of cerebrospinal fluid (CSF) from the paravascular space (VR space) surrounding the small cortical arteries or arterioles to the brain interstitial fluid (ISF) to the paravascular space surrounding veins and venules for drainage to the deep cervical lymphatics. More recent human studies using MRI with CSF tracer demonstrated a similar system and its flow pattern in normal and some disease states (Eide et al., 2018; Ringstad et al., 2018; Davoodi-Bojd et al., 2019; Watts et al., 2019; Taoka and Naganawa, 2021).

## Normal physiological factors in the glymphatic clearance flow system

The functional integrity of the glymphatic system is influenced by several cellular components of the neurovascular unit (Natale et al., 2021). The glymphatic transport system starts with the entry of CSF along the paravascular space, followed by a convective efflux of CSF and admixture with brain ISF. The efflux of CSF into the brain interstitial fluid depends heavily on the function and integrity of the astroglial water channels (Aquaporin-4, AQP4). Impairment in this system is demonstrated with AQP4 knock-out mice, where impaired CSF-ISF movement was shown in AQP4 knock-out mice (Mestre et al., 2018). Without AQP4 channels, a radiotracer in the CSF-ISF mixture accumulated in the brain parenchyma. Its importance and implications in Alzheimer’s disease are also well illustrated in a review by Silva et al. (2021).

The importance of arterial pulsation in driving the glymphatic CSF-ISF flow was also highlighted by Iliff et al. (2013). It was found that a decrease in pulse wave causes a reduction in CSF-ISF exchange (Iliff et al., 2013). Additionally, van Veluw et al. (2020) then demonstrated that vasomotion is an important driving force for paravascular clearance in the awake mouse brain by using the 2-*in vivo* 2-photon microscopy. They also showed that loss of vasomotion due to loss of vascular smooth muscle cells in amyloid angiopathy is associated with impaired solute clearance. Additionally, Drieu et al. (2022) also recently exhibited that there is a population of macrophages in the brain which specifically resides in the perivascular and leptomeningeal areas. These macrophages are strategically positioned next to the CSF, effectively interacting with it and its processes. They called these macrophages parenchymal border macrophages (PBM) and it is evidenced that these macrophages regulate arterial motion that drives CSF flow.

The effect of glymphatic activity on sleep has also been investigated. Given that sleep is when the body does a significant amount of its healing and waste-eliminating processes, it is unsurprising that the glymphatic system was found to be significantly enhanced during sleep. *In vivo* two-photon studies in anesthetized and naturally sleeping mice demonstrated that the sleep state enhances convective fluid fluxes and thereby clearance of metabolites (Xie et al., 2013). Furthermore, aging showed the greatest decline in glymphatic activity in old versus young mice. Studies demonstrated impairment in both influx of CSF tracers and clearance of metabolites (Kress et al., 2014).

In summary, glymphatic flow through the paravascular (VR space) occurs when CSF flows through astroglial AQP4 channels and mixes with ISF to flow toward the venous system where the CSF-ISF mixture enters the paravascular space and is cleared from the brain parenchyma. This flow is dependent on arterial pulsations to create a pressure gradient for the mixture to flow down. Lastly, PBM are important to help regulate the arterial motion and therefore the flow of CSF-ISF.

## Glymphatic flow disturbances in acute stroke including acute SAH and CAA

Gaberel et al. (2014) assessed the integrity of the glymphatic system in four stroke models in mice by using MRI. These include subarachnoid hemorrhage (SAH), intracerebral hemorrhage, carotid ligation, and embolic stroke. They found that the glymphatic function was severely impaired after acute SAH and acute ischemic stroke but not after carotid ligation or intracerebral hemorrhage. It was interesting that glymphatic function improved after intracerebroventricular injection of the fibrinolytic tissue plasminogen activator. Glymphatic perfusion is also restored after arterial recanalization in embolic strokes.

Ringstad et al. (2018) were able to study how the CSF is communicating directly with the extravascular fluid compartment of human brain tissue (ISF) by using MRI with intrathecal injection of the tracer gadobutrol. They found that the gadobutrol tracer enters the brain parenchyma after intrathecal injection into the subarachnoid CSF space as evidenced by brain region contrast enrichment. The tracer enrichment occurred in a centripetal pattern, primarily in regions adjacent to the major pial arterial branches of the anterior, middle, and posterior cerebral arteries, indicating an important role of CSF pulsation for solute transport within the brain parenchyma. They also clearly demonstrated impaired glymphatic tracer clearance in patients with idiopathic normal pressure hydrocephalus. Showing that increased intracranial pressure countered the arterial hydrostatic pressure and further demonstrated the importance of arterial pulsations on for the glymphatic transport system.

Chen et al. (2022) studied the CAA transgenic rat model by using their innovative imaging and analysis tools. They found that CSF moves more rapidly along the para-arterial spaces in transgenic CAA rats. This finding was unexpected given the microvascular deposition and accumulation of Aβ. By further review of the CSF velocity flux vector, they found that CSF currents in CAA are partly diverted from the brain, resulting in impaired glymphatic transport overall. By imaging the cervical lymph nodes, they also found impaired drainage to the deep cervical lymphatics along the carotid arteries, suggesting a concomitant impairment of meningeal lymphatic drainage due to CAA. Most importantly, this study demonstrates that glymphatic flow velocity is actually enhanced in CAA yet the flow at least in part is directed away from the usual direction toward the perivenous areas, resulting in a final impairment of glymphatic clearance.

## Strong evidence of reversed glymphatic flow pattern in a clinical case of acute subarachnoid hemorrhage

In agreement with the study by Chen et al. (2022), we reported a case of relatively limited CAA which developed within a few years after an acute aneurysmal SAH (Lui et al., 2021). This case clearly demonstrated in life the reversal of glymphatic flow vector resulting in subsequent deposition of Aβ in the walls of pial branches of the cerebral arteries.

**FIGURE 1:**
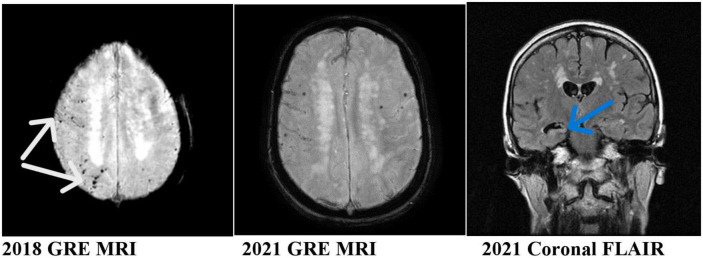
MRI of the patient showing progressive asymmetric (**right** worse than **left**) hemisphere amyloid and hippocampal atrophy. White arrows: microhemorrhages, sites of cerebral amyloid angiopathy. Blue arrow: right hippocampal atrophy.

Our patient is a 59-year-old lady who presented with an acute subarachnoid hemorrhage at the age of 53 in 2016 due to rupture of her right PCoM aneurysm. Her aneurysm was clipped surgically. Since 2020, her family noticed some problems with memory and attention. Her clinical diagnosis was mild cognitive impairment. She had two sets of brain MRI performed in 2018 and 2021. They showed rather striking and progressive development of microhemorrhages as shown in her gradient echo (GRE)/susceptibility-weighted imaging (SWI) MRI. These are lobar microhemorrhages sparing deep hemisphere structures, most compatible with probable amyloid angiopathy according to the modified Boston Criteria (Charidimou et al., 2022; Figure 1). The white arrows in the figure point to the microhemorrhages most compatible with CAA. The MRI appearances are not microhemorrhages due to hypertension because of their locations. It is also not typical for cerebral cavernous malformations without the popcorn appearances in the FLAIR sequence MRI.

Our case is unique and illustrated several important clinicopathological changes which may help in the understanding of the pathogenesis of CAA. She developed apparent progressive clinicopathological changes in her brain after her acute SAH. These include progressive cognitive impairment, progressive right worse than left hemisphere CAA (modified Boston criteria) (Charidimou et al., 2022), and progressive right hippocampal atrophy. Her CAA affected predominantly the brain areas supplied by the right middle cerebral artery, the origin of the posterior communicating artery where her aneurysm ruptured.

From a review of the literature, we know that there is impaired total glymphatic clearance in CAA both in animal models and human studies. However, the glymphatic flow velocity is rapid in CAA. The decreased glymphatic clearance is caused mainly by an abnormal velocity vector, meaning the CSF-ISF flow is directed retrogradely toward the brain surface or cortex in some areas of the brain. This abnormal flow vector can easily be seen in our case of relatively localized CAA after an acute aneurysmal SAH. The Aβ formed in the brain parenchyma will be carried with the CSF-ISF flow toward the small and medium-sized arteries in the Virchow-Robin space and get deposited there. It can easily be visualized that there is extensive fibrin deposition on the surface of the brain. The fibrin will also coat the surface of the penetrating cerebral arteries. As a consequence, the arterial pulsation that propels the CSF-ISF flow is impaired. The arterial vasomotion is also impaired locally. These factors will explain the reduced anterograde glymphatic flow at least from these areas of the brain and overall impaired glymphatic clearance.

**FIGURE 2:**
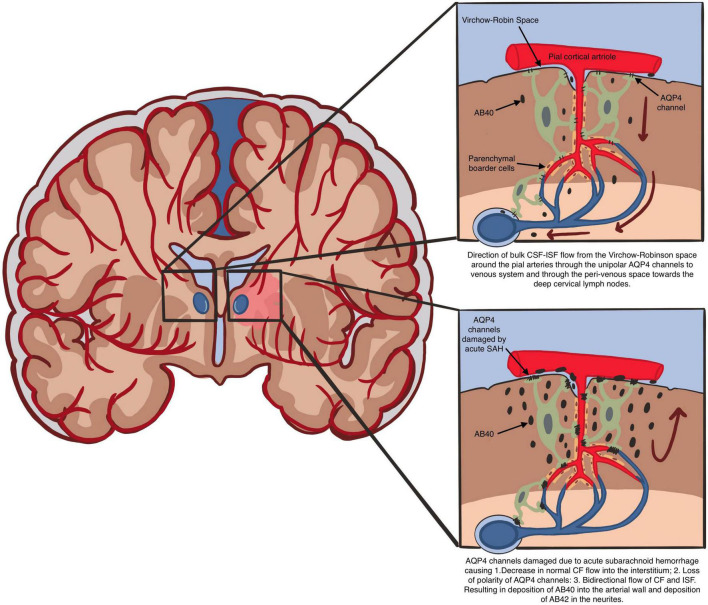
Direction of bulk CSF-ISF flow from the Virchow-Robin space around the pial arteries through the unipolar AQP4 channels to venous system and through the peri-venous space toward the deep cervical lymph nodes. AQP4 channels damaged due to acute subarachnoid hemorrhage causing 1. Decrease in normal CF flow into the interstitium; 2. Loss of polarity of AQP4 channels: 3. Bidirectional flow of CF and ISF. Resulting in deposition of AB40 into the arterial wall and deposition of AB42 in the neurites.

In an attempt to explain the increased glymphatic flow velocity demonstrated by Chen et al. (2022), we have to assume that there is a reversal of CSF-ISF flow in areas of the brain and VR spaces with CAA. This will require damage to the polarity of the AQP4 channels and dysfunction of the parenchymal border macrophages (PBM). This is not unreasonable given the sudden severe rise in the CSF pressure at least locally during acute aneurysmal SAH when the arterial pressure is transmitted to the CSF. It is this reversal of CSF-ISF flow in areas of CAA which will easily explain the increased glymphatic flow velocity associated with ultimate decreased glymphatic clearance (Figure 2).

## Conclusion

The pathogenesis of CAA is the deposition of Aβ in the arterial walls of small and medium-sized arteries and capillaries in the brain parenchyma and leptomeninges. The pathology and progression involve the following processes:

1. Formation of Aβ by the cleavage enzymes acting on the amyloid precursor protein.
2. Disruption of the normal glymphatic flow of CSF-ISF resulting in a reversal of flow in the para-arterial region. This will require a loss of polarity of the AQP4 channels and dysfunction of the PBM.
3. As a result, Aβ will be carried retrogradely and deposited in the small and medium-sized brain parenchymal arteries, affecting the branches of the anterior, middle and posterior cerebral arteries.

Therefore, any therapy for preventing and treating CAA may be directed at preserving the function of AQP4 channels and the parenchymal border macrophages.

## Author contributions

FL prepared and drafted the manuscript. JA, SK, and MY contributed to the literature review, figures drafting, manuscript writing, and editing. NZ critically revised the content and editing of the manuscript. All authors contributed to the article and approved the submitted version.
